# Pneumothorax in the Setting of Spinal Surgery: A Case Report and Review of the Literature

**DOI:** 10.7759/cureus.26743

**Published:** 2022-07-11

**Authors:** Nicole J Levin, Alex R Ghorishi, Neil Charnowitz, Andrew Rosenthal, Jordan Ditchek

**Affiliations:** 1 Medicine, Florida Atlantic University Charles E. Schmidt College of Medicine, Boca Raton, USA; 2 Oral and Maxillofacial Surgery, Memorial Regional Hospital, Hollywood, USA; 3 Trauma Surgery, Memorial Regional Hospital, Hollywood, USA; 4 Radiology, Memorial Regional Hospital, Hollywood, USA

**Keywords:** surgery, surgery complication, trauma, trauma surgery, spinal surgery, pneumothorax

## Abstract

The purpose of this paper is to review the occurrence and management of a tension pneumothorax, which was exacerbated status post posterior spinal surgery. A retrospective review of intraoperative reports, imaging, and pertinent medical records was conducted for a patient who underwent posterior spinal surgery with a tiny apical pneumothorax, which subsequently developed into a major pneumothorax. The clinical signs imperative to recognition and prompt treatment are discussed. Our case report demonstrates that the unrecognized disruption of the pleural cavity during posterior spinal surgery caused the exacerbation of the patient's bilateral pneumothoraces. The patient was successfully treated with finger thoracostomy and chest tube insertion. In conclusion, posterior spinal surgery is an invasive procedure with the potential for serious complications such as the exacerbation of a previous non-surgical pneumothorax. A low index of suspicion is imperative due to the potentially lethal nature of pneumothoraces. Vital signs, pulmonary exam findings, portable radiography, and sonography equipment are all invaluable to the accurate diagnosis and early intervention of patients with pneumothoraces.

## Introduction

A rare but life-threatening complication of posterior spinal surgery is the possible development of a tension pneumothorax, which requires immediate surgical intervention. A simple pneumothorax can evolve into a tension pneumothorax when the patient is under general anesthesia and receiving positive pressure ventilation. In surgery for posterior spinal fixation, pedicle screws are commonly used due to their ability to correct deformities well and their relatively high margin of safety [1]. Current literature has demonstrated that during the placement of pedicle screws, the lateral or ventral walls of the vertebra can be disrupted and may potentially cause the exacerbation of a previous inconsequential pneumothorax [2-4]. Furthermore, if an intraoperative major tension pneumothorax were to develop with the patient in the prone position, the usual sites for pleural punctures and tube insertion would be difficult to access.

Clinical findings associated with the development of a tension pneumothorax include an increase in heart rate, respiratory rate, central venous pressure, airway pressure, and end-tidal carbon dioxide (CO2). Decreased oxygen saturation, hypotension, or tracheal deviation away from the affected side may also occur. However, it is not necessary for all of these physiologic findings to occur for a tension pneumothorax to be present [5,6]. Pneumothorax is readily detected on upright frontal chest radiographs by noting the presence of a “pleural line,” the inwardly displaced and now visualized visceral pleura, and the absence of “lung markings” peripheral to it. Radiographic signs that suggest tension pneumothorax include deviation of the mediastinum toward the contralateral side and depression of the hemidiaphragm on the affected side. Treatment of a tension pneumothorax includes needle decompression with subsequent chest tube placement.

This complication is important to consider during posterior spinal fixation surgery to provide a timely diagnosis and proper management. To date, few studies have acknowledged tension pneumothorax as a complication of posterior spinal surgery. This case report evaluates a trauma patient who underwent spinal surgery for thoracolumbar fractures and had non-surgical tiny apical pneumothoraces develop into major tension pneumothoraces.

## Case presentation

**Figure 1:**
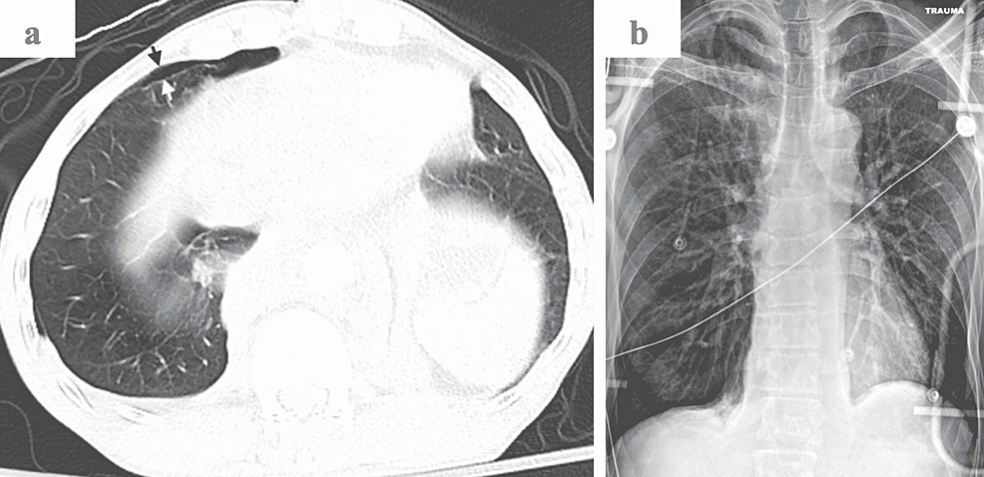
Small right pneumothorax. (A) Axial CT image shows a small amount of air in the pleural space anteriorly, bounded by the visceral pleura (white arrow) and the parietal pleura/chest wall (black arrow). (B) A portable supine chest radiograph fails to demonstrate a pneumothorax of this size.

**Figure 2:**
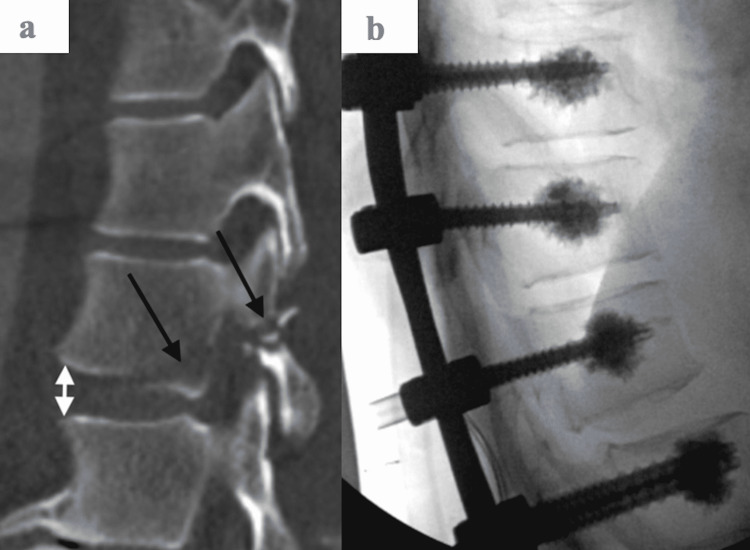
Acute unstable spinal injury. (A) Sagittal CT image shows a fracture of T12, extending through the vertebral body and posterior elements (black arrows). (B) Intraoperative lateral fluoroscopic image shows the placement of posterior fixation instrumentation including bilateral pedicle screws in the lower thoracic spine.

A 52-year-old male presented to the emergency department with a level one trauma status post fall from a 16-foot ladder. The patient did not have any significant past medical history. The patient presented with a Glasgow Coma Scale (GCS) score of 13. The patient endorsed back pain, did not have any sensation below the umbilical region, and was unable to move his lower extremities. Immediate imaging revealed multiple bilateral lower rib fractures, a small right pneumothorax (Figure 1), and small bilateral pleural effusions. Computed tomography (CT) scan (Figure 2) demonstrated fractures of the thoracolumbar spine that were further evaluated by magnetic resonance imaging (MRI).

MRI of the thoracic (T) and lumbar (L) spine without intravenous contrast demonstrated the following injuries: acute unstable fracture with disruption of the anterior and posterior longitudinal ligaments at the T12 to L1 level, a focal tear through the spinal cord at T11, acute compression of T5 and T8 vertebral bodies both with a 25% loss in height, acute fractures of the L1 and L2 spinous processes, left L1, L2, and L3 transverse process fractures, and prevertebral/anterior paraspinal hematomas.

The patient underwent surgery within 24 hours for spine stabilization, spinal cord decompression, and reduction of the fractures. The surgery consisted of T10-L3 posterior instrumentation and fusion with allograft and autograft, T11-L2 laminectomies/osteotomies, reduction of the fracture, cement augmentation of screws, experimental use of bone morphogenic protein, and T8 kyphoplasty (Figure 2).

**Figure 3:**
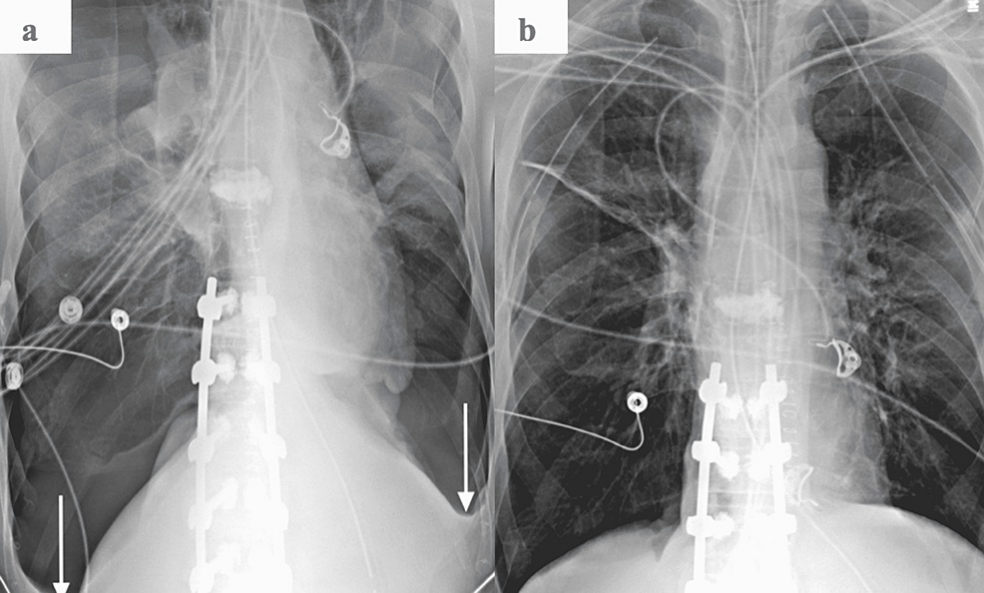
Large bilateral pneumothorax. (A) Supine frontal chest radiograph shows extensive lucency in the lower thorax bilaterally. Note the deep costophrenic sulci (arrows). (B) Portable chest radiograph after bilateral chest tube placement demonstrates re-expansion of both lungs.

A few hours postoperatively, the trauma attending was called to evaluate the patient for severe hypotension and tachycardia. Stat portable chest radiograph demonstrated bilateral pneumothoraces with abnormally deep costophrenic sulci bilaterally (Figure 3). The attending immediately performed bilateral finger thoracostomy and appreciated large rushes of air bilaterally with immediate baseline return of blood pressure. Subsequently, size 28 French chest tubes were inserted bilaterally and secured with 2-0 silk sutures. Both chest tubes were placed to wall-suction and a post-procedure chest X-ray confirmed re-expansion of both lungs (Figure 3).

## Discussion

A pneumothorax is defined as the accumulation of air in the thoracic cavity between the parietal and visceral pleura. Normally during respiration, the intrathoracic cavity is negatively pressurized, allowing the lungs to expand and fill the chest wall. However, in the case of pneumothorax, there is a connection between the thoracic cavity and extra-thoracic space, leading to an influx of air that pushes against and collapses the lung parenchyma. A traumatic pneumothorax develops in the setting of blunt or penetrating trauma, such as a fall from a 16-foot ladder. Positive airway pressures from intraoperative intubation can complicate matters by rapidly increasing accumulated air and intrathoracic pressure. Elevated intrathoracic pressure can not only collapse the lung but can also cause tracheal deviation and depression of the diaphragm. Subsequent hemodynamic instability may develop due to compression of the inferior vena cava leading to decreased venous return and cardiac preload, resulting in obstructive shock manifesting as hypotension, tachycardia, and jugular venous distension [7]. There is an increased risk of progression to cardiorespiratory collapse without prompt thoracostomy. Puncture of the parietal pleura in the context of spinal surgery can further complicate a one-way valve, allowing air to pass into the thoracic cavity with no escape.

Posterior spinal surgery is a significant risk factor for the exacerbation of a previous small pneumothorax, with a 1.6% reported incidence. Multiple intraoperative factors contribute to the risk of pneumothorax, including central line placement, posterior spinal exposure, pedicle screw placement, and posterior rib resections. Surgical teams must be prepared to identify signs and symptoms of pneumothorax both intra- and peri-operatively. Patients are at an increased risk of developing pneumothorax in the post-surgical recovery period due to the closure of the surgical incision site, which disrupts communication between the pleural cavity and the extra-pleural cavity. Surgeons must anticipate potential breaches to the pleura, and chest tube insertion may be warranted prior to closure of the surgical site [8]. In cases of compromised pleural integrity, thoracostomy may be considered even in the absence of hemodynamic changes.

One of the challenges in the detection and management of iatrogenic pneumothorax is the prone positioning of patients during surgery. Radiographic detection of pneumothorax in the prone position is limited [9]. While a chest radiograph obtained with the patient in the upright position has a sensitivity as high as 92% for pneumothorax, alternative positions may only detect pneumothorax in 50% of cases [10]. It is thus imperative that the surgical team can effectively diagnose pneumothorax based on the clinical findings, including increased airway pressure, increased end-tidal CO2 levels, decreased breath sounds on the affected side, decreased tactile fremitus on the affected side, hyperresonance to percussion on the affected side, and changes in heart rate, blood pressure, and oxygen saturation. Furthermore, surgeons should be aware of trauma-associated risk factors for the development of pneumothorax, such as rib fractures and signs of pre-existing pneumothorax, such as subcutaneous emphysema and pulmonary contusions [11].

Future progress for the intraoperative diagnosis of tension pneumothorax includes the possible use of ultrasound in addition to posteroanterior chest radiography. Thoracic ultrasound has demonstrated promising results in the detection and monitoring of pneumothorax during mechanical ventilation in animal models. Ultrasound could therefore represent a simple, cheap, and efficient method for intraoperatively monitoring disturbances to the integrity of the pleural cavity, even in the prone position. Further clinical validation is necessary to determine if ultrasound monitoring improves patient outcomes [12].

Prone positioning not only complicates the diagnosis of pneumothorax but also its treatment. The classical incision site for tension pneumothorax is inaccessible in the prone position. Instead of placing the chest tube in the second intercostal space in the midclavicular line, practitioners may elect to use the fifth intercostal space on the anterior axillary line for emergent thoracostomy [13]. Due to the inherent risks of thoracostomy, its use in the context of spinal surgery is controversial. Ideally, emergent thoracostomy should only be performed in the optimal position if clinically indicated.

## Conclusions

Spinal surgery is an invasive procedure with the potential for serious complications. A low index of suspicion for exacerbation of a simple pneumothorax is imperative as intubation with positive pressure ventilation can cause progression to cardiovascular collapse. Novel methods of intraoperative diagnosis and treatment of pneumothorax in prone positioning, such as ultrasound and lateral needle decompression, are promising options to reduce the morbidity and mortality of posterior spinal surgery. Vital signs, pulmonary exam findings, portable radiography, and sonography equipment are all invaluable to the accurate diagnosis and early intervention of patients with pneumothoraces.
